# Tailored pharmacist-led intervention to improve adherence to Iron supplementation in premature infants: a randomized controlled trial in China

**DOI:** 10.3389/fendo.2023.1288347

**Published:** 2023-10-09

**Authors:** Beimeng Yu, Ming Ni, Haijing Li, Renjie Xu, Aiping Wang

**Affiliations:** ^1^Shaoxing Key Laboratory of Reproductive Health, Shaoxing Maternity and Child Health Care Hospital, Shaoxing, Zhejiang, China; ^2^Neonatal Intensive Care Unit, Shaoxing Maternity and Child Health Care Hospital, Shaoxing, Zhejiang, China; ^3^Department of Clinical Pharmacy, Shaoxing Maternity and Child Health Care Hospital, Shaoxing, Zhejiang, China; ^4^Gynecological Fifth Ward, Shaoxing Maternity and Child Health Care Hospital, Shaoxing, Zhejiang, China

**Keywords:** iron supplementation, genetics, medication adherence, haemoglobin, premature infants

## Abstract

**Introduction:**

Prematurity is due to a number of factors, especially genetics. This study was designed to evaluate the impact of a pharmacist-led patient-centered medication therapy management trial on iron deficiency and medication adherence among premature infants receiving iron supplementation at a tertiary hospital in Shaoxing, China.

**Methods:**

In this randomised controlled trial, eighty-one premature infants, with or without genetic factors, born at 26 to 30 weeks and 6 days gestational age, will be recruited and randomised to an intervention group or a control group. The intervention group will receive a pharmacist-driven discharge counseling on iron supplements from recruitment, until 12 months. The control group will receive care as usual. The main outcomes were haemoglobin (g/L), serum iron (μg/L), medication adherence estimation and differentiation scale, the satisfaction with information about medicines scale, beliefs about medicines questionnaire and the Bayley scales for infant development.

**Results:**

A total of 81 patients were enrolled in the study. After intervention, results for the haemoglobin and serum iron differed significantly between the control group and the intervention group (101.36 vs. 113.55, P < 0.0001 and 51.13 vs. 101.36, P = 0.004). Additionally, there was a substantial difference between the intervention group and the control group in terms of patient medication adherence estimation and differentiation scale (27 vs. 34, P = 0.0002). the intervention group had better mental development index and psychomotor development index, compared with the control group (91.03 vs. 87.29, P = 0.035 and 95.05 vs. 90.00, P = 0.022).

**Discussion:**

In premature infants with iron deficiency, our pharmacist-led team significantly improved clinical outcomes and medication adherence.

## 1 Introduction

Premature birth is caused by many factors, especially genetic factors. Due to limited iron storage brought on by preterm birth, the initiation of postnatal erythropoiesis early, and rapid postpartum growth, premature newborns are at a significant risk of iron shortage (1). Iron is necessary for a wide range of vital processes throughout the development of the brain, and it must be given in sufficient levels, especially to premature infants (2). Premature neonates have fewer iron stores than full-term neonates, according to previous studies, and the smaller they are at birth, the more susceptible they are to iron insufficiency because of their correspondingly lower iron stores (3).

Anemia, low serum ferritin, a high total iron-binding capacity, a high soluble transferrin receptor, and low serum iron are the traditional indicators of iron deficiency (4, 5). Although the exact number of premature infants with an iron deficit cannot be determined, it is anticipated that there will be a high number of these infants given that China has the world’s largest population (6, 7). For a long time, most preterm newborns need to be given a set amount of iron to prevent prematurity anemia, and with iron supplements and acceptable breastfeeding, the condition of iron deficiency gradually improves with age (8). Therefore, effective iron deficiency management, which includes adherence to the iron supplementation, is the key to maximizing the treatment effect in premature infants (9).

The risk of anemia and iron deficiency may be decreased with the optimal adherence to iron supplements. However, a recent 10-year analysis indicated that global medication adherence rates ranged from 3 to 76% (10–12), and that they were between 35 and 48.7% in China (13, 14). Non-adherence may result from a deliberate choice made after weighing the benefits and drawbacks of a drug (intentional non-adherence), from a failure to comprehend the medication regimen or from forgetfulness (unintentional non-adherence) (15, 16). Many pharmacists in China have been released from conventional dispensing as part of the reform of medical policy. One method for changing medication-related perceptions and increasing the adherence to iron supplements treatment among parents of preterm infants is pharmacist-led education and counseling (17, 18).

The purpose of this study was to determine whether one-on-one education and counseling led by a pharmacist could increase iron supplementation adherence and result in changes in hemoglobin levels.

## 2 Materials and methods

### 2.1 Study design and and setting

This was a one year open-label, randomized controlled parallel study conducted in Shaoxing, a typical eastern developed city in China. This observational cohort was established following the Pharmacist-led Patient-Centered Medication Therapy Management trial (ClinicalTrials.gov ID: NCT04740515), a multidisciplinary team’s randomized study of a program to increase compliance among iron deficient patients.

### 2.2 Ethical considerations

The study was approved by the Human Research Ethics Committee at Shaoxing Maternity and Child Health Care Hospital. The 1964 Helsinki declaration and its later amendments, or equivalent ethical standards, were followed in all study protocols. Parental or guardian consent was acquired in writing from each participant.

### 2.3 Recruitment and data collection

**Figure 1:**
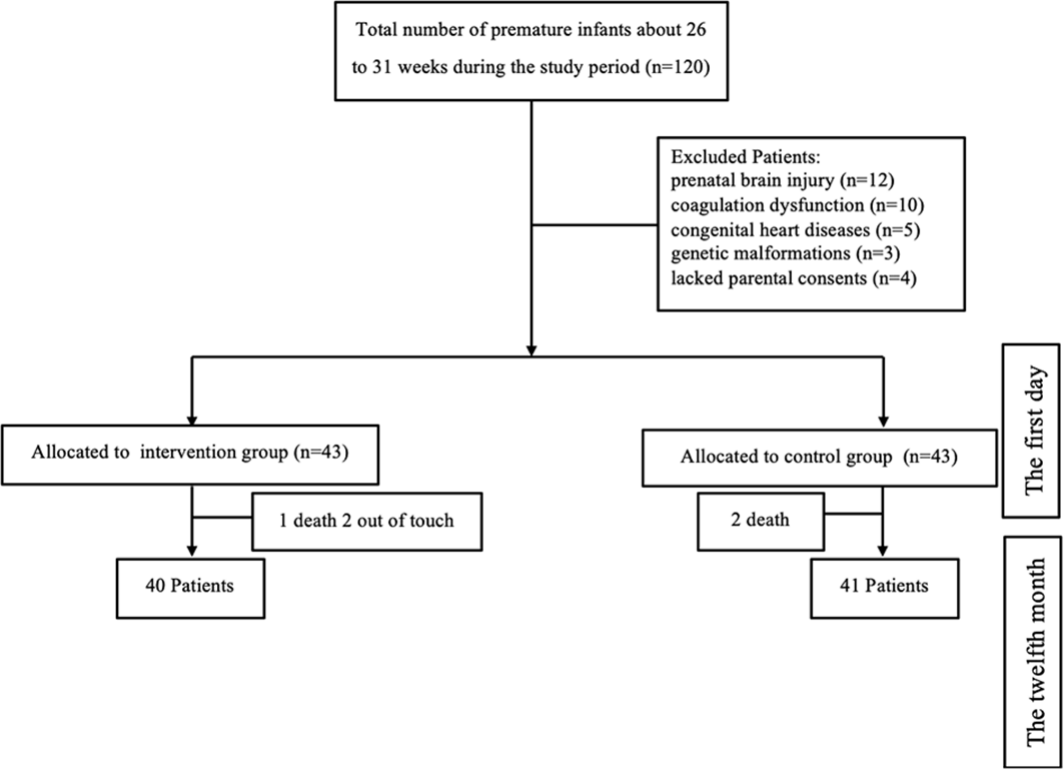
Consort flow diagram of participants in the study.

From Jan 2021 to December 2022, A minimal total sample size of 81 infants born between 26 + ^0^ and 30 + ^6^ weeks of gestation were recruited from Shaoxing maternity and child health care hospitals. In Shaoxing, a representative developed city in eastern China, the hospital is the largest and best-known hospital for the detection and treatment of pediatric illnesses. The study was designed with 80% power (with a 2-sided alpha level of 0.05) to detect a difference of 1 in the mean hemoglobin. The inclusion criteria of premature infants were: (1) a clinically confirmed diagnosis of anemia, and (2) the parents’ written informed consent. The diagnosis of neonatal anemia shall refer to the diagnostic standards formulated in the 《Practical Neonatology》. The exclusion factors included antenatal ultrasonography-detected prenatal brain injury, coagulation abnormalities, congenital cardiac diseases, and genetic abnormalities (**Figure 1**).

### 2.4 Study interventions and follow-up

In addition to usual care, the patient in the intervention group received pharmacist-led multidisciplinary care. The multidisciplinary team consists of a physician, a pharmacist and a nurse. To ensure the uniformity of the material provided during counseling sessions, all professionals attended training before beginning to work with patients. Patients in the control group were given the hospital’s standard of care discharge counseling on iron supplements, whereas patients in the intervention group were given both the standard of care counseling and a pharmacist-driven discharge counseling on iron supplements.

In the intervention group, a pharmacist first spoke with the parents of premature newborns to obtain comprehensive medical, familial, and social histories. A pamphlet with instructions on how to take their medications was given to each parent. Then, they were provided with an educational pamphlet during discharge, Parents signed in the educational pamphlet to confirm that they had received the medication information (**Supporting Information**). The pamphlet contained general advice on lifestyle changes while taking iron supplements, how to handle gastrointestinal reactions if it occurs, general safety measures, and details on over-the-counter medications that might interact with iron supplements. The pamphlet included details on how often to take the medication, management about missing doses, how to store it, how food and medications interact with it, and any other information relevant to iron supplements. Pharmacists explained the content of the pamphlet to the parents, and answered any question related to the drug. All parents in the pharmacist-led group got phone calls from pharmacists as part of the routine clinical follow-up to evaluate their use of iron supplements, to clarify any uncertainties, and to contact their doctors as necessary to address any problems that were discovered. At intervals of one to three months, in conjunction with the dates for clinical follow-up appointments or medication refills, the parents were informed of planned follow-up conversations over the phone or in person. Each visit included a planned, one-on-one education session on medications. In summary, the intervention group added discharge education and follow-up led by pharmacists,

### 2.5 Primary outcome measures

#### 2.5.1 Disease activity

The value of anemia status was shown by haemoglobin (g/L) and serum iron (μg/L). They are generally accepted, valid criteria for measuring disease activity in people with iron deficiency anemia.

#### 2.5.2 Medication adherence assessment

Previous studies show medication adherence estimation and differentiation scale (MEDS) has proven good reliability and validity (19). The MEDS version had 16 items, and each “Never” response received a score of “1,” “Rarely” received a score of “2,” “Sometimes” received a score of “3,” “Often” received a score of “4”, and “Always” received a score of “5”. Consequently, a patient could receive an overall number between 16 (perfectly adherent) and 80 (completely non-adherent).

### 2.6 Secondary outcome measures

#### 2.6.1 Beliefs about medicines questionnaire

The BMQ, which has two portions. BMQ-Specific, which evaluates attitudes about the necessity and concerns for personal medications, and BMQ-General, which evaluates general ideas about overuse and danger of medications, was used to gauge the premature infants’ perception of medication (20). Five items are evaluated on a 5-point Likert scale for each subscale. By deducting the individuals’ worries scores from their requirement scores, a necessity-concerns differential was determined, yielding a range of - 20 to 20 (21).

#### 2.6.2 Satisfaction with information on treatment

The satisfaction with information about medicines measure scale (SIMS) can be used to gauge how satisfied people are with the information given by healthcare professionals like pharmacists. The name of the particular medicine was mentioned in the questionnaire, just like in the BMQ. The amount of information received is rated by parents of premature newborns as “too much,” “about right,” “too little,” “none received,” and “none needed.” For each item, a score is generated in order to determine the parent’s overall level of satisfaction; if the parent is satisfied (responded with “about right”), a score of 1 is provided. This receives a score of 0 if the parent is not satisfied (answers of “too much,” “too little,” “none needed,” or “none received”) 9 of the 17 original questionnaire items that were previously reported were utilized (22). A high score indicates a high level of satisfaction. Scores range from 0 to 9.

#### 2.6.3 Assessment of neurodevelopment

To evaluate mental and motor growth, the second edition of the Bayley Scales for Infant development (BSID-II) was used (23). The BSID offers a mental development index (MDI) and a psychomotor development index (PDI) to measure cognitive abilities. At the hospital, two licensed developmental psychologists with a 90% inter-rater reliability rate administered the BSID at 6 and 12 months. During the evaluations, at least one of the parents was with each child.

### 2.7 Statistical analysis

SPSS 20.0 software was used to evaluate data (SPSS Inc., Chicago, IL, USA). For continuous variables, means and SDs were used to summarize descriptive statistics; for categorical variables, percentages and numbers were used. Categorical variables were analyzed using the χ^2^-test. An independent sample t-test for parametric variables and the Mann-Whitney U-test for non-parametric variables were used to evaluate the relationship between the degree of adherence and continuous variables. The criterion for statistical importance was a p-value of 0.05 or less.

## 3 Results

**Figure 2:**
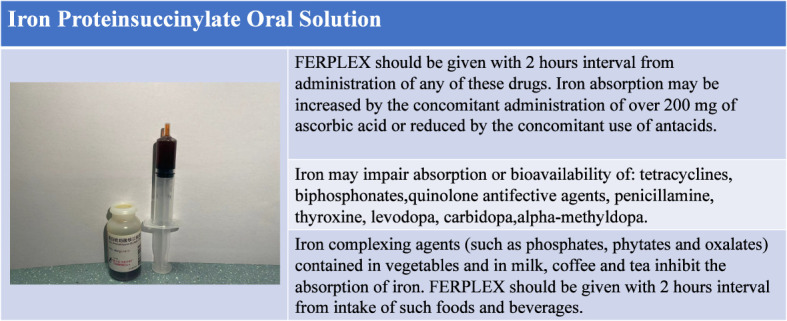
Medication booklet of iron supplementation.

From a total of 120 premature infants enrolled at birth, 86 were randomly assigned to two groups for the intervention, and 81 were eventually evaluated after drop-out rates of 7.4% from birth to 6 months and 6.5% from 6 to 12 months: 40 children in the pharmacist-led multidisciplinary care group and 41 in the non-intervention group (**Figure 2**).

### 3.1 Baseline characteristics

**Table 1** provided details of baseline data or demographic characteristics of the premature infant population. There was no significant difference in any of the baseline parameters. The mean age of the premature infants was both 27.9 weeks respectively, in the intervention group and control group. Of the 81 participants analyzed in the study, 35 (43.2%) were female. Most of the parents were employed (n = 49, 60.5%) and urban residents (n = 64, 79.1%). In terms of education, 7 (17.1%) parents in the control group had a secondary education, and 28 (70%) parents in the intervention group had a high education. In the control group, 19 (46.3%) newborns received blood transfusion during hospitalization, versus 19 (47.5%) in the intervention group. The mean average length of stay of premature infants was 65.5 days in the intervention group and 64.4 days in the control group. In total, 14 premature infants had side effects during the research. Side effects refer to harmful reactions that occur in children during the medication process that are unrelated to the purpose of the medication. No variations in characteristics between the groups were statistically significant at the beginning of the study.

**Table 1:** Baseline characteristics for total randomized premature infants.

Patient characteristics	Control group	Intervention group	P-value
Sex, female, n (%) Gestational age (weeks) Min.–max Mean ± SD Birth weight (kg) Min.–Max Mean ± SD Education level, n (%) Primary (0-9 years) Secondary (9-12 years) Higher (≥13 years) Urban population, n (%) Yes No Employment, n (%) Employed Unemployed Blood transfusion, n (%) Yes No Length of Stay Side effects, n (%) Yes No	18 (43.9) 26-30 27.9 ± 1.36 633-1494 1058.29 ± 298.30 3 (7.3) 7 (17.1) 31 (75.9) 31 (75.6) 10 (24.4) 26 (63.4) 15 (36.6) 19 (46.3) 22 (53.7) 64.4 ± 14.0 6 (14.6) 35 (85.4)	17 (42.5) 26-30 27.9 ± 1.57 634-1367 1006.53 ± 240.54 3 (7.5) 9 (22.5) 28 (70.0) 33 (82.5) 7 (17.5) 23 (57.5) 17 (42.5) 19 (47.5) 21 (52.5) 65.5 ± 11.1 8 (20) 32 (80)	0.899 0.39 0.414 0.455 0.446 0.586 0.917 0.456 0.523

### 3.2 Primary endpoint

The primary endpoint of hemoglobin increase was met in significantly more proportion of premature infants in the intervention group (33.48%) than in the control group (16.19%) at the end of 12 months (p < 0.0001). The mean serum iron was 101.36 ± 8.12 for the intervention group and 51.13 ± 19.53 for the control group (p = 0.004). After 12-month follow up, MEDS scores significantly increased in the intervention group (p = 0.0002) (**Table 2**).

**Table 2:** Primary and secondary outcomes at 12 months.

Outcome	Control group	Intervention group	P-value of difference between groups
**Primary Outcome** **Haemoglobin (g/L)** Initial 12 - month follow-up P-value of difference between time points **Serum Iron (μg/L)** Initial 12 - month follow-up P-value of difference between time points **MEDS scores** Initial 12 - month follow-up P-value of difference between time points in MEDS score **Secondary outcomes** **BMQ score** Initial 12 - month follow-up P-value of difference between time points **SIMS score** Initial 12 - month follow-up P-value of difference between time points **MDI score** 6 - month follow-up 12 - month follow-up P-value of difference between time points **PDI score** 6 - month follow-up 12 - month follow-up P-value of difference between time points	87.24 ± 9.77 101.36 ± 8.12 <0.0001 18.97 ± 9.11 51.13 ± 19.53 <0.0001 42 (26.5~47.0) 34 (25.0~45.0) 0.323 -3 (-4~-1) 1 (-1.6~4) <0.0001 5 (4~-7.5) 6 (4.5~8) 0.29 81.17 ± 8.48 87.29 ± 7.89 <0.0001 83.91 ± 10.08 90.00 ± 10.44 <0.0001	85.07 ± 10.77 113.55 ± 13.53 <0.0001 19.99 ± 8.13 101.36 ± 8.12 <0.0001 41 (30.0~53.0) 27 (21.25~28.75) <0.0001 -2 (-2~1) 2.25 (0.1~5) <0.0001 6 (3.25~-8) 8.5 (6~9) 0.002 82.96 ± 8.54 91.03 ± 8.03 0.001 86.32 ± 9.11 95.05 ± 9.01 0.01	0.345 <0.0001 0.597 0.004 0.505 0.0002 0.26 0.079 0.567 0.004 0.327 0.035 0.265 0.022

### 3.3 Secondary endpoint

In the initial defines about their medications questionnaire, there was no statistically significant difference between the two groups. After 12 months, beliefs scores improved not significantly in the intervention group. A difference was detected between the control and interventional group in the change in SIMS score of at 1.8 points. After monitoring, the newborns’ neurodevelopment status in the intervention group had a significant improvement. The MDI and PDI in the intervention group statistically increased than the control group (91.03 vs 87.29 and 95.05 vs 90.00).

## 4 Discussion

To our knowledge, this was the first study to investigate the pharmacist-led intervention to improve adherence to iron supplementation after birth. In our research, a novel pharmacist-led intervention that included prescription evaluation and modification, patient-centered medical instruction and counseling, and routine follow-up evaluation was given to premature infants who needed iron supplementation. This research demonstrated that educating and counseling dialysis patients about the use of phosphate binders improved their understanding of the medications, increased their sense of the necessity of the treatment, and decreased their concerns. This beneficial outcome was demonstrated by a level of serum iron (101.36 μg/L) and MEDS scores about 27, with statistically significant than control group.

Greater satisfaction with drug information was discovered to be related to the intervention (P = 0.004). Previous research has demonstrated that patient knowledge of medications in people with diabetes (24), hypertension (25) and cancer may be improved by pharmacist-led interdisciplinary care and counseling recommendations (26). Compared to the intervention group, the control group was substantially less satisfied with medication education (6 to 8.5 with P = 0.004). The possible reason is that some premature infants return to the local medical institution after being discharged from our hospital. Parents of these newborns may not have gotten sufficient health education during the follow-up period, which caused their satisfaction with patient education to decline. On the contrary, to ensure that parents in the intervention group got prompt medical consultations, we set up regular meetings or phone calls.

The study team has substantial expertise using the BSID-II to assess neurodevelopment, and both the initial standardization exercises and the quality controls used throughout the study showed strong inter-rater reliability. The BSID-II scores were linked to known risk factors for inadequate neurodevelopment (27), supporting the validity of the test in this research context. In order to ensure accurate measures for these outcomes, the study team underwent a decade of training and standardization in monitoring baby growth. Numerous studies have been conducted on both animal models and newborn humans to determine the relationship between iron deficiency and unfavorable outcomes in the near, medium, and long terms (28–30). With specific areas of impairment connected to brain regions that appeared to be most negatively impacted by iron deficiency, including myelination and the hippocampus. neonatal iron deficiency has been repeatedly linked to inferior neurodevelopmental outcomes overall (31–33). It is particularly important to ensure adequate brain iron status in the neonatal period because, specifically for infants, brain iron deficiency develops more rapidly when there is a lack of iron, and the developmental disruptions brought on by the lack of iron are not reversed by later repletion (34). It can be seen from the results that the neural development of premature infants in the intervention group is better than that in the control group. This may be due to good adherence to iron supplementation, which is a key element in promoting infant neural development. This result also indicates more iron supplementation in the intervention group.

To establish meaningful associations between the results and interventions, A linear regression analysis was done with corresponding 95% confidence intervals, β coefficients for constant, MEDS, haemoglobin, serum iron and SIMS were 2.944 (2,123-3.764), 0.21 (0.013-0.030), -0.12 (-0.018—0.005), -0.007 (-0.012—0.003) and -0.062 (-0.097—0.026), respectively.

In the study, there were a number of limitations. First, this study only included parents who were receiving iron supplements for the first time at a single urban hospital, making it difficult to generalize the results to individuals who were using iron supplements frequently, such as those who had more than two children or resided in rural areas. Second, after initial discharge, some participants might choose to receive follow-up care at other hospitals. Third, because a small percentage of eligible patients declined to participate in the trial, this information was not recorded, making it impossible to report the enrollment rate. Fourth, whereas the other investigator was in charge of postdischarge phone follow-up and iron supplement education, the pharmacist investigator was simply in charge of gathering baseline data and recruiting participants. These pharmacist investigators’ multiple responsibilities could lead to bias (35). Nevertheless, a different researcher who was not involved in daily clinical treatment performed participant randomization, data collection and analysis for haemoglobin (g/L) and serum iron (μg/L), e valuation of medication adherence, and assessment of neurodevelopment.

## 5 Conclusion

Iron is essential for many important processes throughout the entire brain development process. Due to the limited iron storage brought about by premature birth, premature infants face significant risks of iron shortage. For children with anemia caused by iron deficiency, a patient-centered medication management model led by pharmacists can significantly improve the clinical outcomes and medication compliance of children with anemia.

## Data availability statement

The original contributions presented in the study are included in the article/**Supplementary Material**. Further inquiries can be directed to the corresponding author.

## Ethics statement

The studies involving humans were approved by Human Research Ethics Committee at Shaoxing Maternity and Child Health Care Hospital. The studies were conducted in accordance with the local legislation and institutional requirements. Written informed consent for participation in this study was provided by the participants’ legal guardians/next of kin.

## Author contributions

BY: Conceptualization, Writing – original draft. MN: Writing – review & editing. HL: Supervision, Writing – original draft, Formal Analysis. RX: Software, Writing – review & editing. AW: Writing – review & editing.
