# Supplementary material for: Tailored pharmacist-led intervention to improve adherence to Iron supplementation in premature infants: a randomized controlled trial in China
Source: Front Endocrinol (Lausanne). 2023 Oct 9;14:1288347. doi: 10.3389/fendo.2023.1288347 (PMC10591074; doi:10.3389/fendo.2023.1288347)
Supplement: Supplementary file 1 [file Image_1.pdf]

Supplementary Material

Tailored pharmacist-led intervention to improve adherence to Iron supplementation in premature infants: a randomized controlled trial in China

Beimeng Yu,*, Haijing Li, Ming Ni, Renjie Xuand Aiping Wang

*** Correspondence:** Aiping Wang: zjwangaiping@163.com

1 Supplementary Figures and Tables

1.1 Supplementary Figures

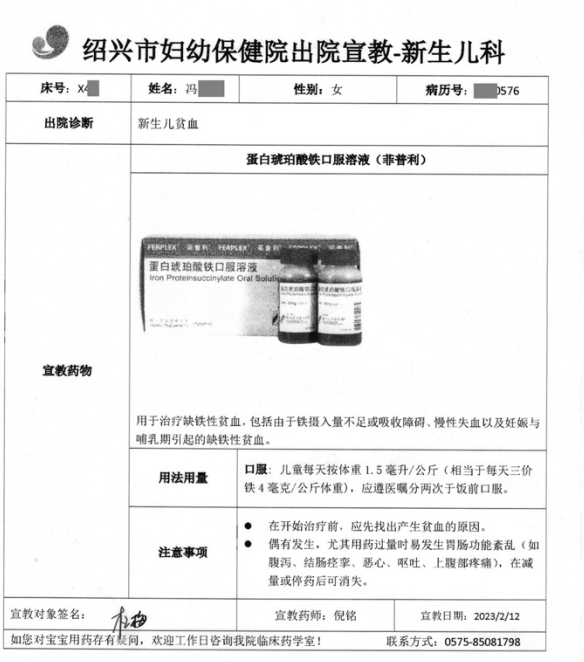

绍兴市妇幼保健院出院宣教-新生儿科

床号：X[redacted]	姓名：冯[redacted]	性别：女	病历号：[redacted]0576
出院诊断	新生儿贫血		
宣教药物	蛋白琥珀酸铁口服溶液（非普利）		
	用于治疗缺铁性贫血，包括由于铁摄入量不足或吸收障碍、慢性失血以及妊娠与哺乳期引起的缺铁性贫血。		
	用法用量	口服：儿童每天按体重 1.5 毫升/公斤（相当于每天三价铁 4 毫克/公斤体重），应遵医嘱分两次于饭前口服。	
	注意事项	● 在开始治疗前，应先找出产生贫血的原因。<br>● 偶有发生，尤其用药过量时易发生胃肠功能紊乱（如腹泻、结肠痉挛、恶心、呕吐、上腹部疼痛），在减量或停药后可消失。	
宣教对象签名：		宣教药师：倪铭	宣教日期：2023/2/12
如您对宝宝用药存有疑问，欢迎工作日咨询我院临床药学室！			联系方式：0575-85081798

Supplementary Figure 1. Educational pamphlet of iron supplementation for premature infants
